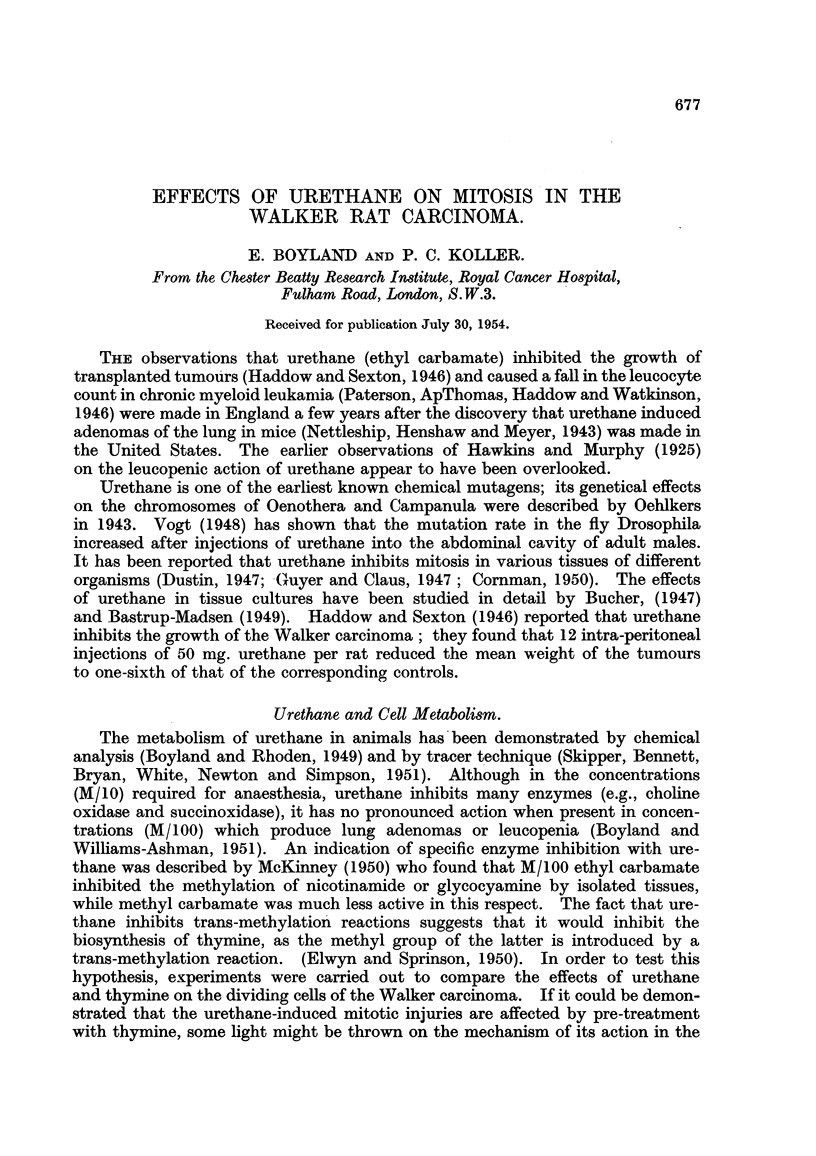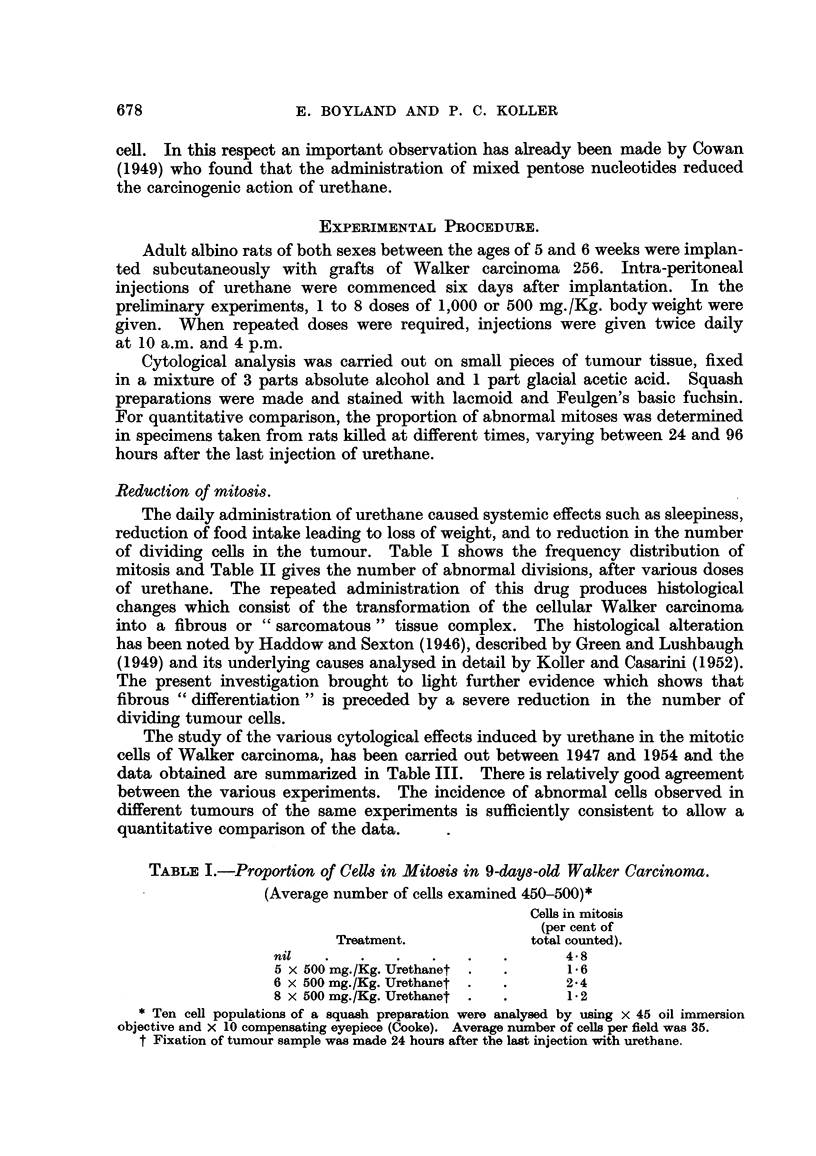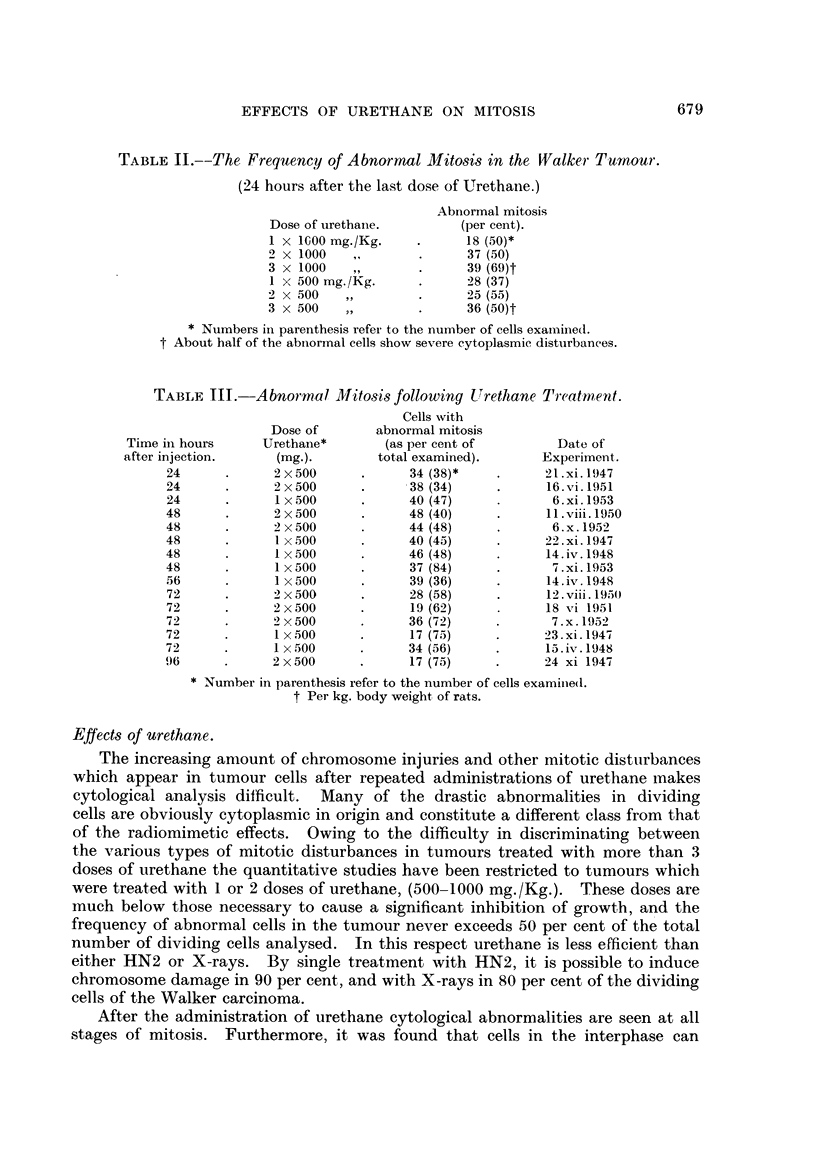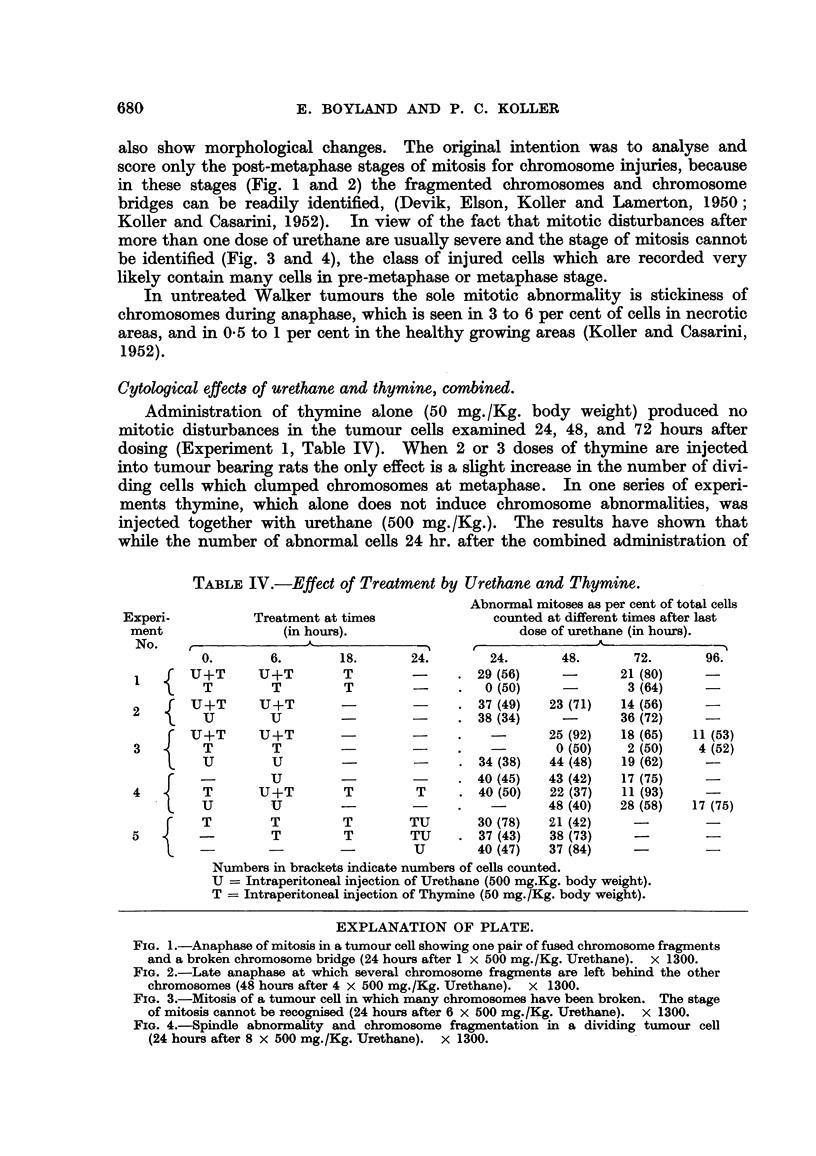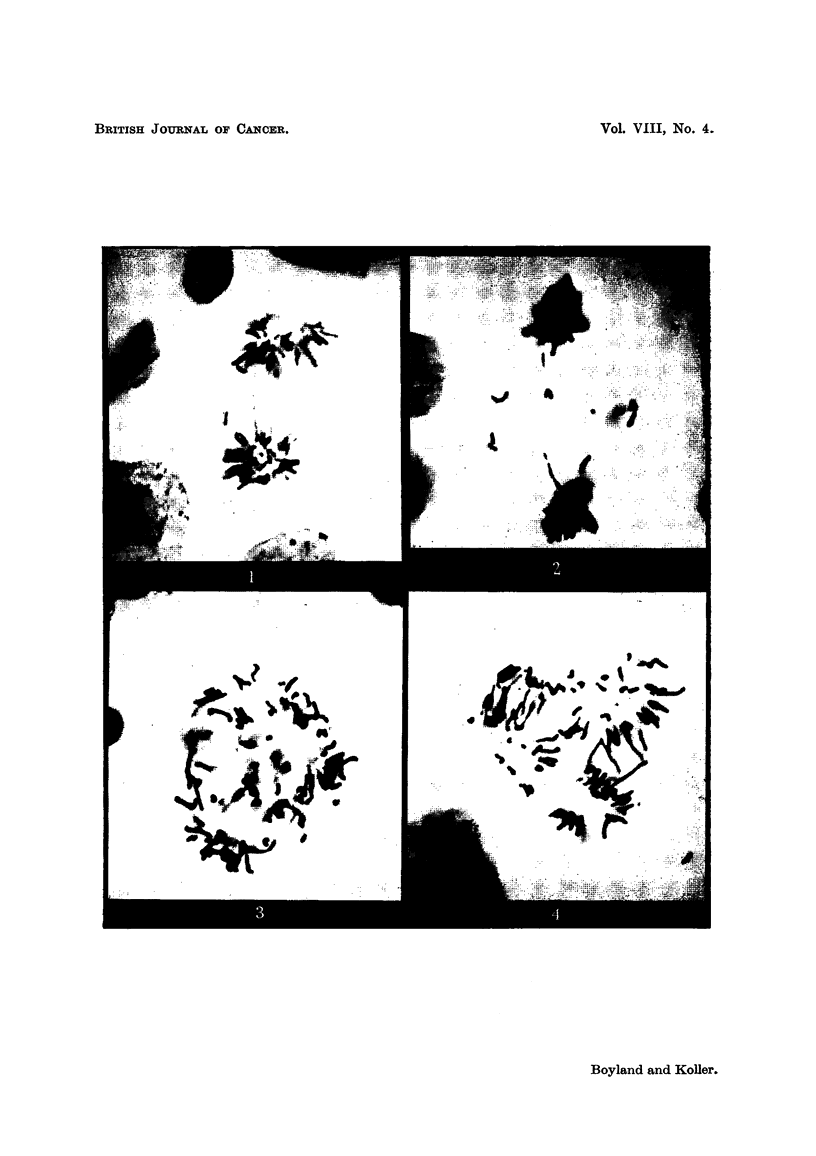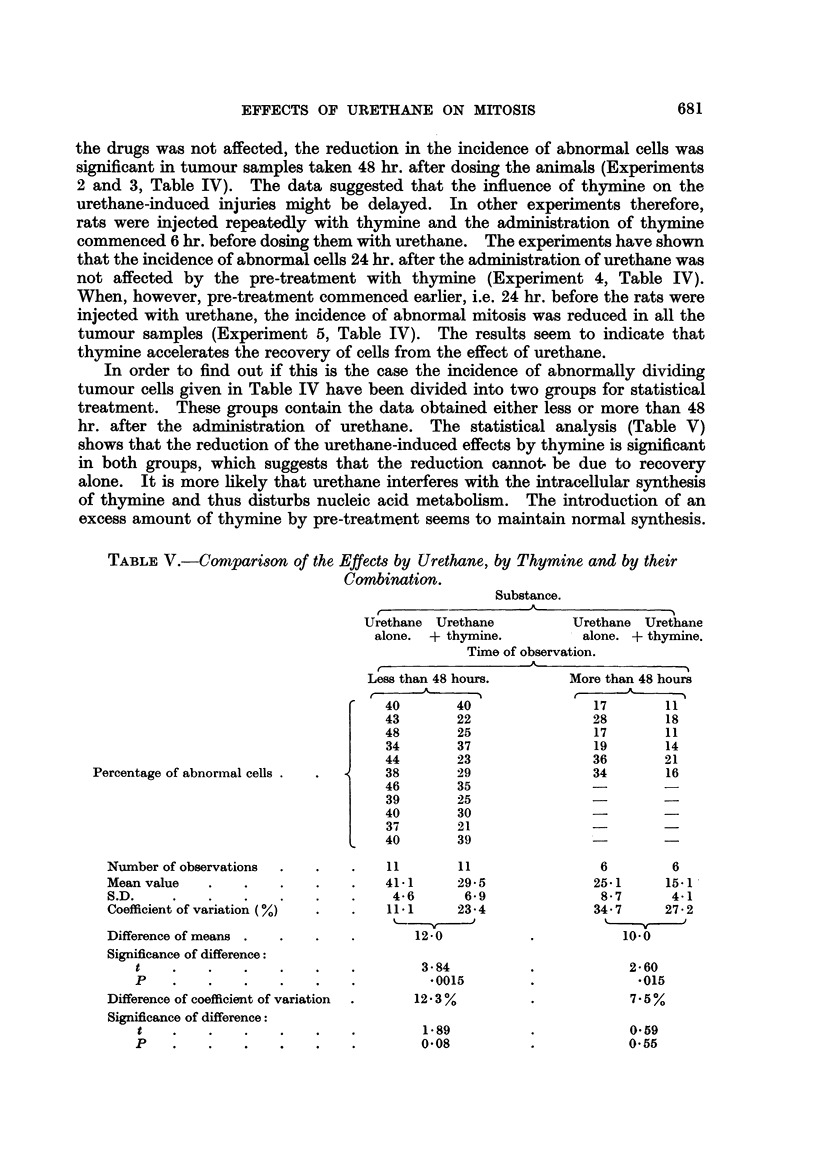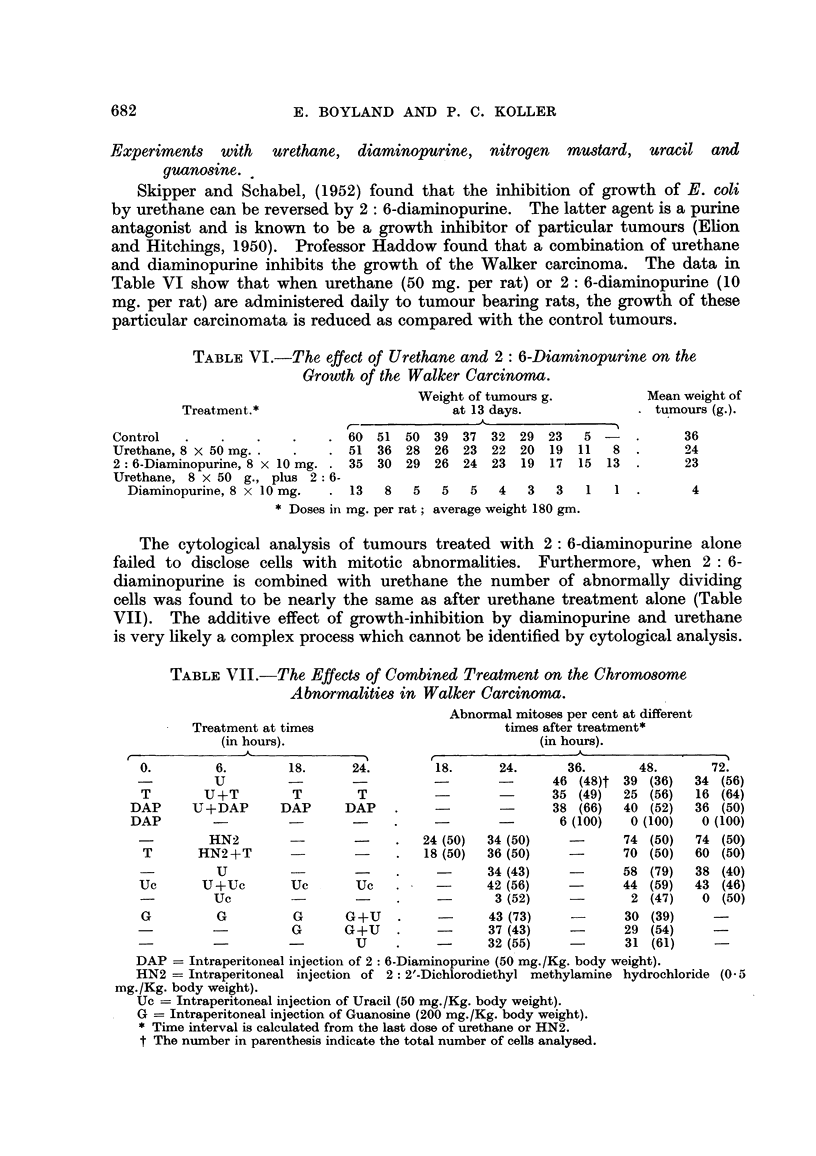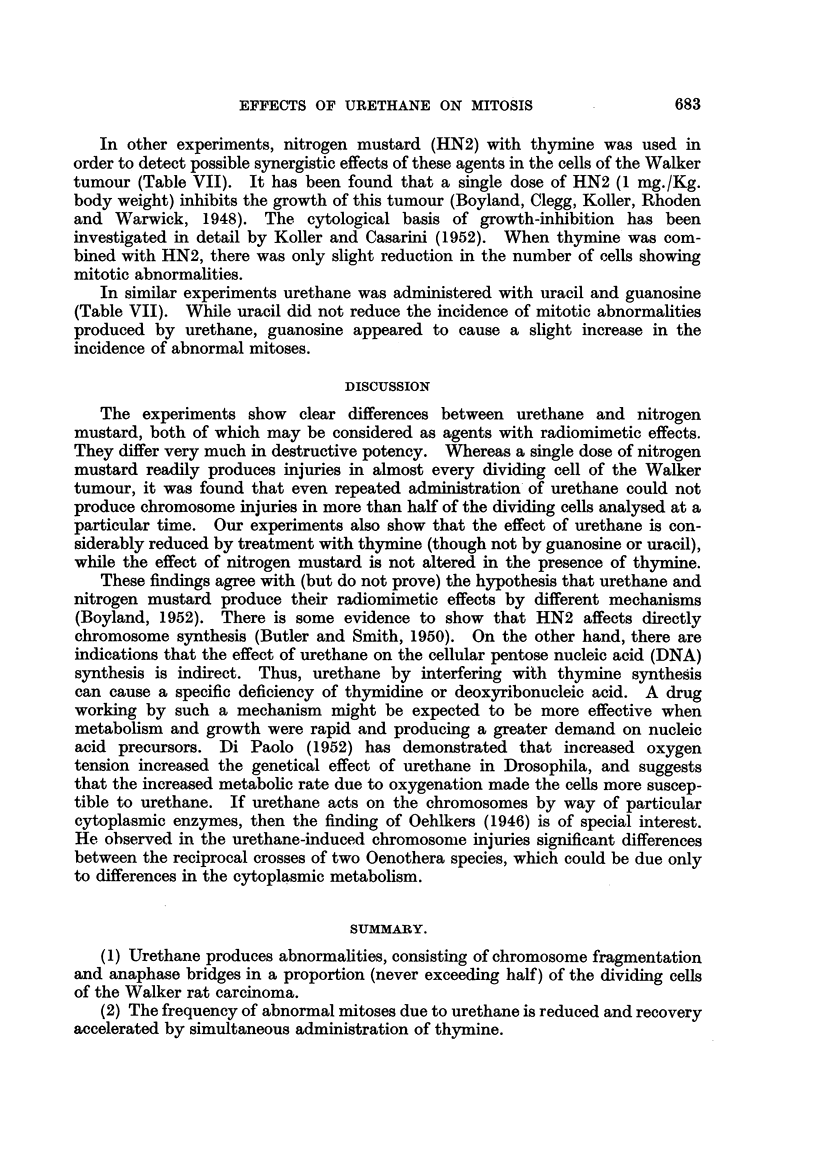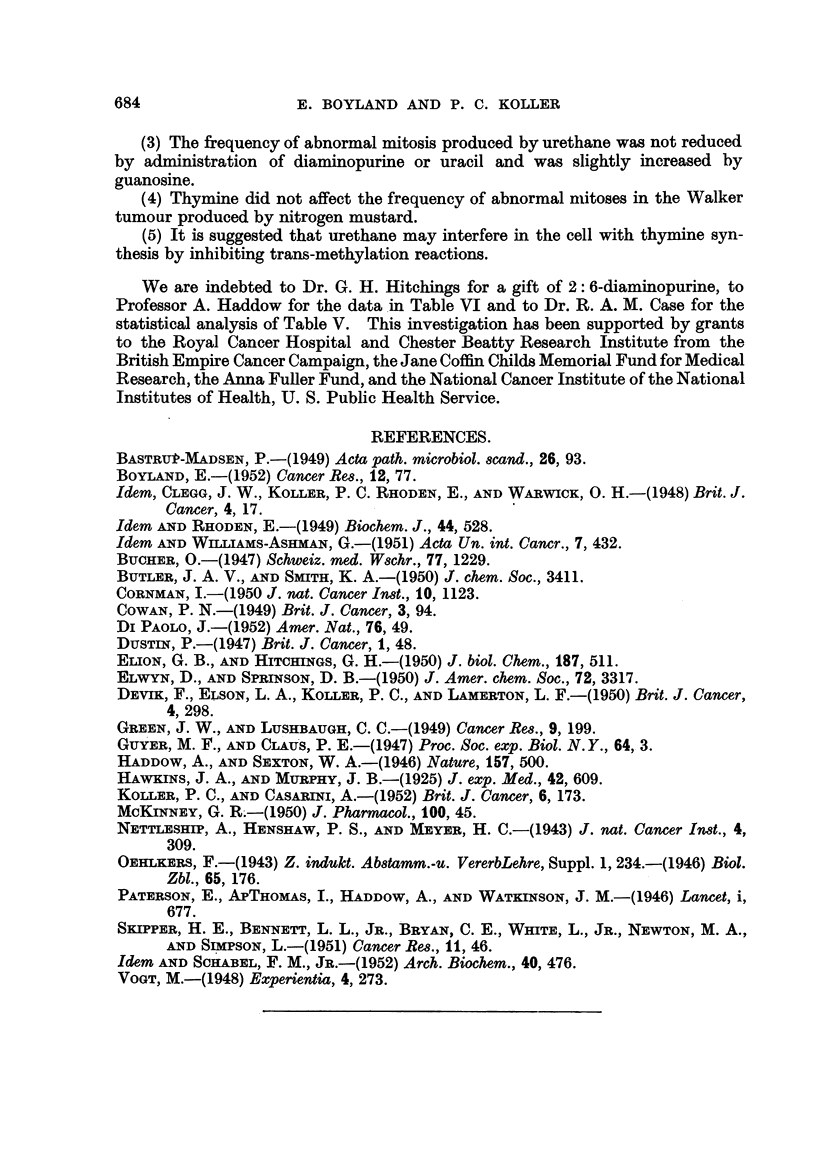# Effects of Urethane on Mitosis in the Walker Rat Carcinoma

**DOI:** 10.1038/bjc.1954.73

**Published:** 1954-12

**Authors:** E. Boyland, P. C. Koller

## Abstract

**Images:**


### EFFECTS OF URETHANE ON MITOSIS IN THE WALKER RAT CARCINOMA.

E. BOYLAND AND P. C. KOLLER.

*From the Chester Beatty Research Institute, Royal Cancer Hospital, Fulham Road, London, S.W.3.*

Received for publication July 30, 1954.

THE observations that urethane (ethyl carbamate) inhibited the growth of transplanted tumours (Haddow and Sexton, 1946) and caused a fall in the leucocyte count in chronic myeloid leukamia (Paterson, ApThomas, Haddow and Watkinson, 1946) were made in England a few years after the discovery that urethane induced adenomas of the lung in mice (Nettleship, Henshaw and Meyer, 1943) was made in the United States. The earlier observations of Hawkins and Murphy (1925) on the leucopenic action of urethane appear to have been overlooked.

Urethane is one of the earliest known chemical mutagens; its genetical effects on the chromosomes of Oenothera and Campanula were described by Oehlkers in 1943. Vogt (1948) has shown that the mutation rate in the fly Drosophila increased after injections of urethane into the abdominal cavity of adult males. It has been reported that urethane inhibits mitosis in various tissues of different organisms (Dustin, 1947; Guyer and Claus, 1947 ; Cornman, 1950). The effects of urethane in tissue cultures have been studied in detail by Bucher, (1947) and Bastrup-Madsen (1949). Haddow and Sexton (1946) reported that urethane inhibits the growth of the Walker carcinoma ; they found that 12 intra-peritoneal injections of 50 mg. urethane per rat reduced the mean weight of the tumours to one-sixth of that of the corresponding controls.

### *Urethane and Cell Metabolism.*

The metabolism of urethane in animals has been demonstrated by chemical analysis (Boyland and Rhoden, 1949) and by tracer technique (Skipper, Bennett, Bryan, White, Newton and Simpson, 1951). Although in the concentrations (M/10) required for anaesthesia, urethane inhibits many enzymes (e.g., choline oxidase and succinoxidase), it has no pronounced action when present in concentrations (M/100) which produce lung adenomas or leucopenia (Boyland and Williams-Ashman, 1951). An indication of specific enzyme inhibition with urethane was described by McKinney (1950) who found that M/100 ethyl carbamate inhibited the methylation of nicotinamide or glycocyamine by isolated tissues, while methyl carbamate was much less active in this respect. The fact that urethane inhibits trans-methylation reactions suggests that it would inhibit the biosynthesis of thymine, as the methyl group of the latter is introduced by a trans-methylation reaction. (Elwyn and Sprinson, 1950). In order to test this hypothesis, experiments were carried out to compare the effects of urethane and thymine on the dividing cells of the Walker carcinoma. If it could be demonstrated that the urethane-induced mitotic injuries are affected by pre-treatment with thymine, some light might be thrown on the mechanism of its action in the cell. In this respect an important observation has already been made by Cowan (1949) who found that the administration of mixed pentose nucleotides reduced the carcinogenic action of urethane.

### EXPERIMENTAL PROCEDURE.

Adult albino rats of both sexes between the ages of 5 and 6 weeks were implanted subcutaneously with grafts of Walker carcinoma 256. Intra-peritoneal injections of urethane were commenced six days after implantation. In the preliminary experiments, 1 to 8 doses of 1,000 or 500 mg./Kg. body weight were given. When repeated doses were required, injections were given twice daily at 10 a.m. and 4 p.m.

Cytological analysis was carried out on small pieces of tumour tissue, fixed in a mixture of 3 parts absolute alcohol and 1 part glacial acetic acid. Squash preparations were made and stained with lacmoid and Feulgen's basic fuchsin. For quantitative comparison, the proportion of abnormal mitoses was determined in specimens taken from rats killed at different times, varying between 24 and 96 hours after the last injection of urethane.

*Reduction of mitosis.*

The daily administration of urethane caused systemic effects such as sleepiness, reduction of food intake leading to loss of weight, and to reduction in the number of dividing cells in the tumour. Table I shows the frequency distribution of mitosis and Table II gives the number of abnormal divisions, after various doses of urethane. The repeated administration of this drug produces histological changes which consist of the transformation of the cellular Walker carcinoma into a fibrous or "sarcomatous" tissue complex. The histological alteration has been noted by Haddow and Sexton (1946), described by Green and Lushbaugh (1949) and its underlying causes analysed in detail by Koller and Casarini (1952). The present investigation brought to light further evidence which shows that fibrous "differentiation" is preceded by a severe reduction in the number of dividing tumour cells.

The study of the various cytological effects induced by urethane in the mitotic cells of Walker carcinoma, has been carried out between 1947 and 1954 and the data obtained are summarized in Table III. There is relatively good agreement between the various experiments. The incidence of abnormal cells observed in different tumours of the same experiments is sufficiently consistent to allow a quantitative comparison of the data.

TABLE I.—*Proportion of Cells in Mitosis in 9-days-old Walker Carcinoma.*

(Average number of cells examined 450–500)*

| Treatment. | Cells in mitosis (per cent of total counted). |
|---|---|
| *nil* | 4·8 |
| 5 × 500 mg./Kg. Urethane† | 1·6 |
| 6 × 500 mg./Kg. Urethane† | 2·4 |
| 8 × 500 mg./Kg. Urethane† | 1·2 |

* Ten cell populations of a squash preparation were analysed by using × 45 oil immersion objective and × 10 compensating eyepiece (Cooke). Average number of cells per field was 35.

† Fixation of tumour sample was made 24 hours after the last injection with urethane.

TABLE II.—*The Frequency of Abnormal Mitosis in the Walker Tumour.*
(24 hours after the last dose of Urethane.)

| Dose of urethane. | Abnormal mitosis (per cent). |
|---|---|
| 1 × 1000 mg./Kg. | 18 (50)* |
| 2 × 1000 ,, | 37 (50) |
| 3 × 1000 ,, | 39 (69)† |
| 1 × 500 mg./Kg. | 28 (37) |
| 2 × 500 ,, | 25 (55) |
| 3 × 500 ,, | 36 (50)† |

* Numbers in parenthesis refer to the number of cells examined.
† About half of the abnormal cells show severe cytoplasmic disturbances.

TABLE III.—*Abnormal Mitosis following Urethane Treatment.*

| Time in hours after injection. | Dose of Urethane* (mg.). | Cells with abnormal mitosis (as per cent of total examined). | Date of Experiment. |
|---|---|---|---|
| 24 | 2×500 | 34 (38)* | 21.xi.1947 |
| 24 | 2×500 | 38 (34) | 16.vi.1951 |
| 24 | 1×500 | 40 (47) | 6.xi.1953 |
| 48 | 2×500 | 48 (40) | 11.viii.1950 |
| 48 | 2×500 | 44 (48) | 6.x.1952 |
| 48 | 1×500 | 40 (45) | 22.xi.1947 |
| 48 | 1×500 | 46 (48) | 14.iv.1948 |
| 48 | 1×500 | 37 (84) | 7.xi.1953 |
| 56 | 1×500 | 39 (36) | 14.iv.1948 |
| 72 | 2×500 | 28 (58) | 12.viii.1950 |
| 72 | 2×500 | 19 (62) | 18 vi 1951 |
| 72 | 2×500 | 36 (72) | 7.x.1952 |
| 72 | 1×500 | 17 (75) | 23.xi.1947 |
| 72 | 1×500 | 34 (56) | 15.iv.1948 |
| 96 | 2×500 | 17 (75) | 24 xi 1947 |

* Number in parenthesis refer to the number of cells examined.
† Per kg. body weight of rats.

*Effects of urethane.*

The increasing amount of chromosome injuries and other mitotic disturbances which appear in tumour cells after repeated administrations of urethane makes cytological analysis difficult. Many of the drastic abnormalities in dividing cells are obviously cytoplasmic in origin and constitute a different class from that of the radiomimetic effects. Owing to the difficulty in discriminating between the various types of mitotic disturbances in tumours treated with more than 3 doses of urethane the quantitative studies have been restricted to tumours which were treated with 1 or 2 doses of urethane, (500–1000 mg./Kg.). These doses are much below those necessary to cause a significant inhibition of growth, and the frequency of abnormal cells in the tumour never exceeds 50 per cent of the total number of dividing cells analysed. In this respect urethane is less efficient than either HN2 or X-rays. By single treatment with HN2, it is possible to induce chromosome damage in 90 per cent, and with X-rays in 80 per cent of the dividing cells of the Walker carcinoma.

After the administration of urethane cytological abnormalities are seen at all stages of mitosis. Furthermore, it was found that cells in the interphase can also show morphological changes. The original intention was to analyse and score only the post-metaphase stages of mitosis for chromosome injuries, because in these stages (Fig. 1 and 2) the fragmented chromosomes and chromosome bridges can be readily identified, (Devik, Elson, Koller and Lamerton, 1950; Koller and Casarini, 1952). In view of the fact that mitotic disturbances after more than one dose of urethane are usually severe and the stage of mitosis cannot be identified (Fig. 3 and 4), the class of injured cells which are recorded very likely contain many cells in pre-metaphase or metaphase stage.

In untreated Walker tumours the sole mitotic abnormality is stickiness of chromosomes during anaphase, which is seen in 3 to 6 per cent of cells in necrotic areas, and in 0·5 to 1 per cent in the healthy growing areas (Koller and Casarini, 1952).

*Cytological effects of urethane and thymine, combined.*

Administration of thymine alone (50 mg./Kg. body weight) produced no mitotic disturbances in the tumour cells examined 24, 48, and 72 hours after dosing (Experiment 1, Table IV). When 2 or 3 doses of thymine are injected into tumour bearing rats the only effect is a slight increase in the number of dividing cells which clumped chromosomes at metaphase. In one series of experiments thymine, which alone does not induce chromosome abnormalities, was injected together with urethane (500 mg./Kg.). The results have shown that while the number of abnormal cells 24 hr. after the combined administration of

TABLE IV.—*Effect of Treatment by Urethane and Thymine.*

| Experiment No. | Treatment at times (in hours). | | | | Abnormal mitoses as per cent of total cells counted at different times after last dose of urethane (in hours). | | | |
|---|---|---|---|---|---|---|---|---|
| | 0. | 6. | 18. | 24. | 24. | 48. | 72. | 96. |
| 1 | U+T | U+T | T | — | 29 (56) | — | 21 (80) | — |
| | T | T | T | — | 0 (50) | — | 3 (64) | — |
| 2 | U+T | U+T | — | — | 37 (49) | 23 (71) | 14 (56) | — |
| | U | U | — | — | 38 (34) | — | 36 (72) | — |
| 3 | U+T | U+T | — | — | — | 25 (92) | 18 (65) | 11 (53) |
| | T | T | — | — | — | 0 (50) | 2 (50) | 4 (52) |
| | U | U | — | — | 34 (38) | 44 (48) | 19 (62) | — |
| 4 | — | U | — | — | 40 (45) | 43 (42) | 17 (75) | — |
| | T | U+T | T | T | 40 (50) | 22 (37) | 11 (93) | — |
| | U | U | — | — | — | 48 (40) | 28 (58) | 17 (75) |
| 5 | T | T | T | TU | 30 (78) | 21 (42) | — | — |
| | — | T | T | TU | 37 (43) | 38 (73) | — | — |
| | — | — | — | U | 40 (47) | 37 (84) | — | — |

Numbers in brackets indicate numbers of cells counted.
U = Intraperitoneal injection of Urethane (500 mg.Kg. body weight).
T = Intraperitoneal injection of Thymine (50 mg./Kg. body weight).

### EXPLANATION OF PLATE.

FIG. 1.—Anaphase of mitosis in a tumour cell showing one pair of fused chromosome fragments and a broken chromosome bridge (24 hours after 1 × 500 mg./Kg. Urethane). × 1300.

FIG. 2.—Late anaphase at which several chromosome fragments are left behind the other chromosomes (48 hours after 4 × 500 mg./Kg. Urethane). × 1300.

FIG. 3.—Mitosis of a tumour cell in which many chromosomes have been broken. The stage of mitosis cannot be recognised (24 hours after 6 × 500 mg./Kg. Urethane). × 1300.

FIG. 4.—Spindle abnormality and chromosome fragmentation in a dividing tumour cell (24 hours after 8 × 500 mg./Kg. Urethane). × 1300.

Boyland and Koller.

the drugs was not affected, the reduction in the incidence of abnormal cells was significant in tumour samples taken 48 hr. after dosing the animals (Experiments 2 and 3, Table IV). The data suggested that the influence of thymine on the urethane-induced injuries might be delayed. In other experiments therefore, rats were injected repeatedly with thymine and the administration of thymine commenced 6 hr. before dosing them with urethane. The experiments have shown that the incidence of abnormal cells 24 hr. after the administration of urethane was not affected by the pre-treatment with thymine (Experiment 4, Table IV). When, however, pre-treatment commenced earlier, i.e. 24 hr. before the rats were injected with urethane, the incidence of abnormal mitosis was reduced in all the tumour samples (Experiment 5, Table IV). The results seem to indicate that thymine accelerates the recovery of cells from the effect of urethane.

In order to find out if this is the case the incidence of abnormally dividing tumour cells given in Table IV have been divided into two groups for statistical treatment. These groups contain the data obtained either less or more than 48 hr. after the administration of urethane. The statistical analysis (Table V) shows that the reduction of the urethane-induced effects by thymine is significant in both groups, which suggests that the reduction cannot be due to recovery alone. It is more likely that urethane interferes with the intracellular synthesis of thymine and thus disturbs nucleic acid metabolism. The introduction of an excess amount of thymine by pre-treatment seems to maintain normal synthesis.

TABLE V.—*Comparison of the Effects by Urethane, by Thymine and by their Combination.*

| | Substance. | | | |
|---|---|---|---|---|
| | Urethane alone. | Urethane + thymine. | Urethane alone. | Urethane + thymine. |
| | Time of observation. | | | |
| | Less than 48 hours. | | More than 48 hours | |
| Percentage of abnormal cells | 40 | 40 | 17 | 11 |
| | 43 | 22 | 28 | 18 |
| | 48 | 25 | 17 | 11 |
| | 34 | 37 | 19 | 14 |
| | 44 | 23 | 36 | 21 |
| | 38 | 29 | 34 | 16 |
| | 46 | 35 | — | — |
| | 39 | 25 | — | — |
| | 40 | 30 | — | — |
| | 37 | 21 | — | — |
| | 40 | 39 | — | — |
| Number of observations | 11 | 11 | 6 | 6 |
| Mean value | 41·1 | 29·5 | 25·1 | 15·1 |
| S.D. | 4·6 | 6·9 | 8·7 | 4·1 |
| Coefficient of variation (%) | 11·1 | 23·4 | 34·7 | 27·2 |
| Difference of means | 12·0 | | 10·0 | |
| Significance of difference: | | | | |
| *t* | 3·84 | | 2·60 | |
| *P* | ·0015 | | ·015 | |
| Difference of coefficient of variation | 12·3% | | 7·5% | |
| Significance of difference: | | | | |
| *t* | 1·89 | | 0·59 | |
| *P* | 0·08 | | 0·55 | |

*Experiments with urethane, diaminopurine, nitrogen mustard, uracil and guanosine.*

Skipper and Schabel, (1952) found that the inhibition of growth of *E. coli* by urethane can be reversed by 2 : 6-diaminopurine. The latter agent is a purine antagonist and is known to be a growth inhibitor of particular tumours (Elion and Hitchings, 1950). Professor Haddow found that a combination of urethane and diaminopurine inhibits the growth of the Walker carcinoma. The data in Table VI show that when urethane (50 mg. per rat) or 2 : 6-diaminopurine (10 mg. per rat) are administered daily to tumour bearing rats, the growth of these particular carcinomata is reduced as compared with the control tumours.

TABLE VI.—*The effect of Urethane and 2 : 6-Diaminopurine on the Growth of the Walker Carcinoma.*

| Treatment.* | Weight of tumours g. at 13 days. | | | | | | | | | | Mean weight of tumours (g.). |
|---|---|---|---|---|---|---|---|---|---|---|---|
| Control | 60 | 51 | 50 | 39 | 37 | 32 | 29 | 23 | 5 | — | 36 |
| Urethane, 8 × 50 mg. | 51 | 36 | 28 | 26 | 23 | 22 | 20 | 19 | 11 | 8 | 24 |
| 2 : 6-Diaminopurine, 8 × 10 mg. | 35 | 30 | 29 | 26 | 24 | 23 | 19 | 17 | 15 | 13 | 23 |
| Urethane, 8 × 50 g., plus 2 : 6-Diaminopurine, 8 × 10 mg. | 13 | 8 | 5 | 5 | 5 | 4 | 3 | 3 | 1 | 1 | 4 |

* Doses in mg. per rat ; average weight 180 gm.

The cytological analysis of tumours treated with 2 : 6-diaminopurine alone failed to disclose cells with mitotic abnormalities. Furthermore, when 2 : 6-diaminopurine is combined with urethane the number of abnormally dividing cells was found to be nearly the same as after urethane treatment alone (Table VII). The additive effect of growth-inhibition by diaminopurine and urethane is very likely a complex process which cannot be identified by cytological analysis.

TABLE VII.—*The Effects of Combined Treatment on the Chromosome Abnormalities in Walker Carcinoma.*

| Treatment at times (in hours). 0. | 6. | 18. | 24. | Abnormal mitoses per cent at different times after treatment* (in hours). 18. | 24. | 36. | 48. | 72. |
|---|---|---|---|---|---|---|---|---|
| — | U | — | — | — | — | 46 (48)† | 39 (36) | 34 (56) |
| T | U+T | T | T | — | — | 35 (49) | 25 (56) | 16 (64) |
| DAP | U+DAP | DAP | DAP | — | — | 38 (66) | 40 (52) | 36 (50) |
| DAP | — | — | — | — | — | 6 (100) | 0 (100) | 0 (100) |
| — | HN2 | — | — | 24 (50) | 34 (50) | — | 74 (50) | 74 (50) |
| T | HN2+T | — | — | 18 (50) | 36 (50) | — | 70 (50) | 60 (50) |
| — | U | — | — | — | 34 (43) | — | 58 (79) | 38 (40) |
| Uc | U+Uc | Uc | Uc | — | 42 (56) | — | 44 (59) | 43 (46) |
| — | Uc | — | — | — | 3 (52) | — | 2 (47) | 0 (50) |
| G | G | G | G+U | — | 43 (73) | — | 30 (39) | — |
| — | — | G | G+U | — | 37 (43) | — | 29 (54) | — |
| — | — | — | U | — | 32 (55) | — | 31 (61) | — |

DAP = Intraperitoneal injection of 2 : 6-Diaminopurine (50 mg./Kg. body weight).
HN2 = Intraperitoneal injection of 2 : 2′-Dichlorodiethyl methylamine hydrochloride (0·5 mg./Kg. body weight).
Uc = Intraperitoneal injection of Uracil (50 mg./Kg. body weight).
G = Intraperitoneal injection of Guanosine (200 mg./Kg. body weight).
* Time interval is calculated from the last dose of urethane or HN2.
† The number in parenthesis indicate the total number of cells analysed.

In other experiments, nitrogen mustard (HN2) with thymine was used in order to detect possible synergistic effects of these agents in the cells of the Walker tumour (Table VII). It has been found that a single dose of HN2 (1 mg./Kg. body weight) inhibits the growth of this tumour (Boyland, Clegg, Koller, Rhoden and Warwick, 1948). The cytological basis of growth-inhibition has been investigated in detail by Koller and Casarini (1952). When thymine was combined with HN2, there was only slight reduction in the number of cells showing mitotic abnormalities.

In similar experiments urethane was administered with uracil and guanosine (Table VII). While uracil did not reduce the incidence of mitotic abnormalities produced by urethane, guanosine appeared to cause a slight increase in the incidence of abnormal mitoses.

### DISCUSSION

The experiments show clear differences between urethane and nitrogen mustard, both of which may be considered as agents with radiomimetic effects. They differ very much in destructive potency. Whereas a single dose of nitrogen mustard readily produces injuries in almost every dividing cell of the Walker tumour, it was found that even repeated administration of urethane could not produce chromosome injuries in more than half of the dividing cells analysed at a particular time. Our experiments also show that the effect of urethane is considerably reduced by treatment with thymine (though not by guanosine or uracil), while the effect of nitrogen mustard is not altered in the presence of thymine.

These findings agree with (but do not prove) the hypothesis that urethane and nitrogen mustard produce their radiomimetic effects by different mechanisms (Boyland, 1952). There is some evidence to show that HN2 affects directly chromosome synthesis (Butler and Smith, 1950). On the other hand, there are indications that the effect of urethane on the cellular pentose nucleic acid (DNA) synthesis is indirect. Thus, urethane by interfering with thymine synthesis can cause a specific deficiency of thymidine or deoxyribonucleic acid. A drug working by such a mechanism might be expected to be more effective when metabolism and growth were rapid and producing a greater demand on nucleic acid precursors. Di Paolo (1952) has demonstrated that increased oxygen tension increased the genetical effect of urethane in Drosophila, and suggests that the increased metabolic rate due to oxygenation made the cells more susceptible to urethane. If urethane acts on the chromosomes by way of particular cytoplasmic enzymes, then the finding of Oehlkers (1946) is of special interest. He observed in the urethane-induced chromosome injuries significant differences between the reciprocal crosses of two Oenothera species, which could be due only to differences in the cytoplasmic metabolism.

### SUMMARY.

(1) Urethane produces abnormalities, consisting of chromosome fragmentation and anaphase bridges in a proportion (never exceeding half) of the dividing cells of the Walker rat carcinoma.

(2) The frequency of abnormal mitoses due to urethane is reduced and recovery accelerated by simultaneous administration of thymine.

(3) The frequency of abnormal mitosis produced by urethane was not reduced by administration of diaminopurine or uracil and was slightly increased by guanosine.

(4) Thymine did not affect the frequency of abnormal mitoses in the Walker tumour produced by nitrogen mustard.

(5) It is suggested that urethane may interfere in the cell with thymine synthesis by inhibiting trans-methylation reactions.

We are indebted to Dr. G. H. Hitchings for a gift of 2 : 6-diaminopurine, to Professor A. Haddow for the data in Table VI and to Dr. R. A. M. Case for the statistical analysis of Table V. This investigation has been supported by grants to the Royal Cancer Hospital and Chester Beatty Research Institute from the British Empire Cancer Campaign, the Jane Coffin Childs Memorial Fund for Medical Research, the Anna Fuller Fund, and the National Cancer Institute of the National Institutes of Health, U. S. Public Health Service.

### REFERENCES.

BASTRUP-MADSEN, P.—(1949) *Acta path. microbiol. scand.*, **26**, 93.
BOYLAND, E.—(1952) *Cancer Res.*, **12**, 77.
*Idem*, CLEGG, J. W., KOLLER, P. C. RHODEN, E., AND WARWICK, O. H.—(1948) *Brit. J. Cancer*, **4**, 17.
*Idem* AND RHODEN, E.—(1949) *Biochem. J.*, **44**, 528.
*Idem* AND WILLIAMS-ASHMAN, G.—(1951) *Acta Un. int. Cancr.*, **7**, 432.
BUCHER, O.—(1947) *Schweiz. med. Wschr.*, **77**, 1229.
BUTLER, J. A. V., AND SMITH, K. A.—(1950) *J. chem. Soc.*, 3411.
CORNMAN, I.—(1950 *J. nat. Cancer Inst.*, **10**, 1123.
COWAN, P. N.—(1949) *Brit. J. Cancer*, **3**, 94.
DI PAOLO, J.—(1952) *Amer. Nat.*, **76**, 49.
DUSTIN, P.—(1947) *Brit. J. Cancer*, **1**, 48.
ELION, G. B., AND HITCHINGS, G. H.—(1950) *J. biol. Chem.*, **187**, 511.
ELWYN, D., AND SPRINSON, D. B.—(1950) *J. Amer. chem. Soc.*, **72**, 3317.
DEVIK, F., ELSON, L. A., KOLLER, P. C., AND LAMERTON, L. F.—(1950) *Brit. J. Cancer*, **4**, 298.
GREEN, J. W., AND LUSHBAUGH, C. C.—(1949) *Cancer Res.*, **9**, 199.
GUYER, M. F., AND CLAUS, P. E.—(1947) *Proc. Soc. exp. Biol. N.Y.*, **64**, 3.
HADDOW, A., AND SEXTON, W. A.—(1946) *Nature*, **157**, 500.
HAWKINS, J. A., AND MURPHY, J. B.—(1925) *J. exp. Med.*, **42**, 609.
KOLLER, P. C., AND CASARINI, A.—(1952) *Brit. J. Cancer*, **6**, 173.
McKINNEY, G. R.—(1950) *J. Pharmacol.*, **100**, 45.
NETTLESHIP, A., HENSHAW, P. S., AND MEYER, H. C.—(1943) *J. nat. Cancer Inst.*, **4**, 309.
OEHLKERS, F.—(1943) *Z. indukt. Abstamm.-u. VererbLehre*, Suppl. 1, 234.—(1946) *Biol. Zbl.*, **65**, 176.
PATERSON, E., APTHOMAS, I., HADDOW, A., AND WATKINSON, J. M.—(1946) *Lancet*, i, 677.
SKIPPER, H. E., BENNETT, L. L., JR., BRYAN, C. E., WHITE, L., JR., NEWTON, M. A., AND SIMPSON, L.—(1951) *Cancer Res.*, **11**, 46.
*Idem* AND SCHABEL, F. M., JR.—(1952) *Arch. Biochem.*, **40**, 476.
VOGT, M.—(1948) *Experientia*, **4**, 273.